# Occupational Risks Associated with Solid Waste Management in the Informal Sector of Gweru, Zimbabwe

**DOI:** 10.1155/2016/9024160

**Published:** 2016-06-21

**Authors:** Steven Jerie

**Affiliations:** Department of Geography and Environmental Studies, Midlands State University, P. Bag 9055, Gweru, Zimbabwe

## Abstract

This study identifies and analyses the occupational risks associated with solid waste management practices in the informal enterprises of Gweru. Many concerns have been raised about the potential harm from waste to the environment and the general public, but the risks and consequent costs of occupational hazards in waste management have received little attention in the rush to adopt or adapt technologies such as composting. A multimethods research design that triangulates qualitative and quantitative research paradigms is employed in this study. The quantitative design involves physical characterisation of solid waste through material component separation and measurements as well as a questionnaire survey that investigates the risks associated with waste management. The qualitative component includes interviews, open-ended questionnaires, and field observations. Occupational risks occur at every stage in the waste management process, from the point where workers handle waste in the enterprises for collection or recycling to the point of ultimate disposal. Key findings from the study revealed that solid waste management practices are dominated by manual handling tasks hence the higher incidents of muscular-skeletal disorders. Other safety and health hazards associated with waste management in the informal enterprises of Gweru include incidents of diarrhoea, viral hepatitis, and higher incidents of obstructive and restrictive disorders.

## 1. Introduction

Protection of human health and the environment is one of the major challenges facing developing as well as developed countries of the world [1–5]. The original aim of regulating waste disposal is to reduce the introduction of polluting substances into the atmosphere since protection of the environment is a major challenge facing developing countries such as Zimbabwe. The activities in solid waste management in the informal enterprises of Gweru involve risk either to the worker directly involved or to the informal enterprise operators. Risks occur at every stage in the process, from the point where enterprise operators handle waste in their enterprises for collection or recycling to the point of ultimate disposal [6–11]. The informal sector enterprise activities generate large quantities of waste which could be detrimental not only to the environment but to the waste workers as well. Many concerns have been raised about the potential harm from waste to the environment and general public, but the risks and consequent costs of occupational hazards in waste management have received little attention in the rush to adopt or adapt technologies such as composting. Environmental policies and legislation are, in the main, aimed at regulating the disposal of waste rather than addressing and preventing its generation. In some countries of the developed world attention seems to have shifted towards policies and legislation designed to minimise the generation of waste and to secure its beneficial reutilisation. It is therefore vital in this study to examine the occupational safety and health hazards associated with solid waste generated and disposed of in the informal enterprises of the city of Gweru the third largest urban settlement in Zimbabwe.

The city of Gweru covers an area of about 16 700 square kilometres and lies in Agro-Ecological Region Three. The main types of soils that characterise the landscape of Gweru include black basalt soils, red loams, sands, and gravel. It lies in a watershed which stretches from Rusape to Bulawayo and is at an altitude of 1422 meters. The municipal area is dissected by numerous streams most of which drain into the Gweru river, a tributary of Gwayi river. Gweru's annual rainfall averages 500–750 mm, characterised by midseason dry spells. The normal rainy season starts in October right through to April. In the past few years, however, the rainfall patterns have been increasingly becoming poor, with seasons, in some cases, ending in March. Temperatures are high in summer (September to April) when they may average 30°C and low in winter (May to July) averaging 14°C. Gweru comprises low, medium, and high density residential areas. Mkoba high density suburb located in the western section of the city of Gweru is the largest low income residential area and is divided into 20 sections which are referred to as “villages.” Other low income high density suburbs include Ascot, Monomotapa, Senga-Nehosho, and Mambo and the middle density suburbs include Ivene, Woodlands, Nashville, and Shamrock Park. High income low density areas are located mainly in the northern and eastern sectors of the city and include Lundi Park, Southdowns, Kopje, Gweru East, Windsor Park, Daylesford, Harben Park, Brackenhurst, Ridgemond, Athlone, and Riverside. The settlements in Gweru are divided into 18 wards and these are located in different directions from the CBD. According to the 2012 National Census of Zimbabwe undertaken by ZIMSTAT, the total population of Gweru was 158 233 comprising 73 768 males and 84 465 females and the households make a total of 41 149 out of the city's 18 wards. The suburbs or residential areas are divided into wards for ease of administration and service provision by the local council.

The city of Gweru has always had great potential for growth due to its endowment with a wide variety of industries and as a result it has the highest per capita income in Zimbabwe. This has resulted in it being the city with the highest employment rate per capita in the country. It is centrally located and hence it is a very accessible place and the hub of traffic traversing the country. The major industries include Zimbabwe Alloys, a chrome smelting plant, and Bata Shoe Company (established in 1939), and both are leading employers in Gweru. The city is situated in one of Zimbabwe's finest cattle rearing areas: the surrounding agricultural activity revolves around the cattle industry (both beef and dairy). Flowers are also grown in the area for the export market, and Zimbabwe's largest distiller, Afdis, has extensive vineyards in Gweru for the production of wine. Mining is also prevalent, mainly chromite ore from rich deposits along the Great Dyke to the east of Gweru.

The objective of any informal enterprise should be to minimise the amounts of unwanted products or by-products so as to reduce impact on human health and the environment [13–19]. It is vital to examine the potential environmental and health impacts of waste generated in the informal enterprises since this gives an indication of the effectiveness of the waste management practices such as waste collection and disposal. According to Tchobanoglous et al. [12] hazardous wastes are wastes or combinations of waste that pose a substantial present or potential hazard to humans or other living organisms because such wastes are nonbiodegradable or persistent in nature; they can be biologically magnified, can be lethal, or may otherwise cause or tend to cause detrimental cumulative effects. The Environmental Management Act of Zimbabwe defines a hazardous substance as any substance whether solid, liquid, or gas or any organism which is injurious to human health or the environment. It further defines hazardous waste as any waste which is poisonous, corrosive, noxious, explosive, inflammable, radioactive, toxic, or harmful to the environment. The properties of solid waste that have been used to assess whether the waste is hazardous or not are related to questions of safety (corrosivity, explosivity, flammability, ignitability, and reactivity) and health (carcinogenicity, infectivity, irritability, mutagenicity, toxicity, radioactivity, and teratogenicity).

There has been growing concern over the disposal of solid waste, which may contain small amounts of hazardous waste. Hazardous products generated in the informal enterprises, just like those generated in the domestic and industrial sectors, pose a threat to human health and the environment in their use and disposal. In this study, the amounts of hazardous waste in the informal sector solid waste stream were determined by measuring components separated mechanically (by hand) from the commingled waste. Wastes become a problem when they are harmful to the environment or human health and in this regard the wastes become hazardous. The shrinking of the formal sector industries in Zimbabwe has resulted in the growth of home industries in the city of Gweru. These home industries generate solid waste and sound management of the waste is the greatest challenge currently facing these industries. These activities produce high quantities of waste which could be detrimental to the health of the waste worker and environment by contributing to air, water, and land pollution as well as pollution of the visual environment and hence a number of safety and health risks if the waste is not properly managed through an efficient waste management system. The informal enterprises are recognised as part of a waste management system in an urban environment in terms of waste recycling. Studies in Zimbabwe have made preliminary assessments on the impact of domestic and formal waste on the environment [20–24], but no comprehensive study has been made to determine the characteristics of waste generated in the informal enterprises as well as the occupational safety and health hazards associated with the collection and disposal of the waste. Studies have not clearly articulated the issue of the occupational safety and health risks of waste generation, collection, and disposal in the informal enterprises of Zimbabwe as deserving investigation because some say it is difficult to study and probably the government does not directly generate any revenue from this sector. This is an area in which our ignorance exceeds our knowledge and hence deserves special attention in this study.

## 2. Methodology

**Figure 1:**
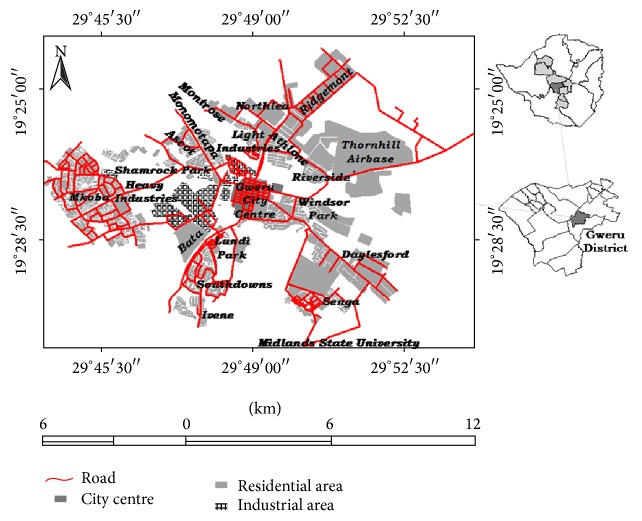
Location of informal enterprises in the city of Gweru.

The research design employed in the study was closely related to the ontological and epistemological assumptions held about reality by the various stakeholders associated with the informal enterprises of Gweru. A multimethods approach triangulating quantitative (for generating hard data) and qualitative (for generating soft data) approaches was thus employed in the study area comprising a sample of 601 informal enterprises. In this study working procedures, conditions, and occupational safety and health risks were assessed in the informal enterprises of Gweru. The study population for questionnaire surveys comprised all the 589 organised informal enterprises in Monomotapa high density suburb, Shamrock Park medium density suburb, Mkoba high density suburb, Ascot high density suburb, Kudzanai market, and Kombayi market. The location of these enterprises in Gweru is shown in Figure 1. Focus was on these areas because of the large concentrations of informal enterprises characterised by a diverse range of enterprises that include retail, service, repair, manufacturing, and construction activities. In Monomotapa 47 out of 51 enterprises agreed to participate in the survey. At Shamrock Park there was a combination of informal enterprises and small-scale and medium-scale enterprises. All the 57 informal sector enterprises were selected to participate in the survey and they were those with less than 10 employees and the small-scale and medium-scale enterprises were left out since they did not meet the criteria for defining informal enterprises. All the 182 enterprises at Kudzanai that were allocated with stalls from which they operated were involved in this study and participated with keen interest and the majority are retailers of food and clothing. The other market area near the city centre is at Kombayi and all the 29 informal enterprises that were allocated stalls participated in the study and at Kudzanai these are mainly food and clothing retailers. In Mkoba and Ascot high density suburbs the majority of enterprise operators participated in the survey and the very few that declined to participate were either suspicious or simply uncooperative. Out of a total of 229 enterprises in Mkoba, 224 participated from the sections of Mkoba 6, Mkoba 14, and Mkoba 16 and in Ascot a total of 50 out of 53 enterprises participated in the study. All in all 589 enterprises participated in the questionnaire survey.

Questionnaire surveys were used to realise the immediate objectives of the research as well as to gather data on the informal enterprises of Gweru. To gather data on critical areas of solid waste management in the informal sector, the design as recommended by Oppenheim [25], De Vaus [26], and Baker [27] was used so as to reduce ambiguity or bias. The questionnaire was developed to cover aspects of the objectives to investigate issues concerning informal enterprise waste generation and disposal practices, availability and type of waste disposal services, and perceptions on the waste management situation in the informal enterprises and how the system can be improved. The questionnaire administered to the home industry operators aimed at collecting information on the quantity and type of waste produced, waste collection and disposal practices, and the occupational safety and health hazards associated with these activities. The instrument was divided into appropriate sections to allow for the systematic collection of data from the enterprises in the different spatial locations of Monomotapa, Shamrock Park, Mkoba, Kudzanai, Kombayi market, and Ascot. The survey questionnaire was semistructured, containing both open-ended and closed-ended questions. Interviews were for the purpose of gathering information on waste management system in Gweru's home industries, occupational safety and health problems associated with solid waste management, planning for waste management in informal enterprises and environmental impact of waste produced in the home industries. The interviews targeted policy makers and planners in the organisations dealing with waste management. Personal observations were undertaken to assess the typical tasks performed during waste collection and disposal and the associated hazards. In addition to the field assessments a focus group discussion was held in each of the spatial areas was held. This was meant to assess the perceptions of the workers as well as enterprise operators on risk perception, injuries and diseases linked to waste occupation and their own ideas for improvement options. Data collection for the waste compositional study followed the traditional material based classification adopted by Burnley [28]. The samples from the informal enterprises were collected in plastic bags and labelled with unique identity marks. The segregated components were weighed to determine weights as percentages of total weight of a sample.

## 3. Results and Analysis

### 3.1. Solid Waste Generation in the Informal Sector of Gweru

#### 3.1.1. Composition of General Waste

It is generally assumed that solid waste generated in the informal enterprises contributes an insignificant proportion to the total waste stream generated in any urban environment and hence it does not deserve special attention. However, the study reveals that significant quantities of solid waste are generated in the informal sector of Gweru especially in market areas that focus on retailing of vegetable and food products and the industrial sectors involved in manufacturing and construction. The major components of the waste stream include food and vegetable wastes at Monomotapa, Ascot, and Mkoba (51%, 29%, and 18% of total weight, resp.), metals at Shamrock Park, Monomotapa, and Mkoba (36%, 31%, and 19% of total weight, resp.), and paper at Mkoba, Ascot, and Kudzanai (11%, 11%, and 9% of total weight, resp.). Solid waste generated in the retail sector is dominated by biodegradable waste in the form of food and vegetable waste as well as long-term biodegradable (incinerable) wastes such as paper, textiles, rubber, and leather products. The biodegradable waste stream dominates in the market areas of Kudzanai and Kombayi where it constitutes an average of 57.1% of waste generated in these areas. In the market areas located in Ascot and Mkoba, the biodegradable fraction comprises 31.6% and 20%, respectively, of the waste generated in those areas. It is important to note that biodegradability is a vital biological characteristic of the organic component of solid waste. Therefore, wastes with low lignin content such as food wastes and vegetable wastes are more biodegradable than those with high lignin content such as paper and plastic that are dominant in some enterprises. Establishing biodegradability of solid waste is essential because the majority of environmental and health problems associated with waste generated in the enterprises are caused by the biodegradable components. This assertion confirms findings in the literature regarding the impacts of biodegradability of solid waste on human health and the environment (see [29–35] and Tchobanoglous, 2003).

The nonbiodegradable waste fraction includes metals, plastics, and inerts arising out of builder's rubble. Metals dominate in the manufacturing and construction enterprises at Monomotapa and Shamrock Park and constitute on average 30.6% and 39.6%, respectively, of the total waste generated in those areas. This can be attributed to the nature of activities associated with these enterprises that include welding, steel fabrication, panel beating, mechanical and electrical engineering, and tinsmithing. The dry recyclables such as paper, plastics, and glass are lower in most cases due to the informal practices of waste reduction, reuse, and recycling with the involvement of rag pickers, itinerant buyers, and dealers of recyclables.

#### 3.1.2. The Composition of Hazardous Waste

The composition of hazardous waste generated in the informal enterprises of Gweru is shown in Table 1. Hazardous waste contributes on average 2.6% of total waste by weight in the informal enterprises. Although occurring in small quantities, the hazardous solid waste can have significant negative impacts on human health and environment when improperly disposed of. The hazardous wastes pose substantial present or potential hazards to humans or other living organisms because they are nondegradable, are persistent in nature, or are lethal. The typical problems associated with hazardous enterprise wastes identified above are summarised in Table 2. In the informal enterprises of Gweru, the hazardous waste stream comprises mainly cleaning products, personal care products, automotive products, pesticides, insecticides, and herbicides, and miscellany which incorporates batteries and sharps such as broken glassware.

There is risk caused by the myriad of toxic chemicals present in some the hazardous waste shown in Table 2, especially the e-waste because of its association with heavy metals such as arsenic, cadmium, chromium, lead, and mercury. These heavy metals have no beneficial effects in humans and there is no known homeostasis mechanism for them. These elements are regarded as most toxic to humans and animals and the adverse human health effects associated with exposure to them, even at low concentrations, are adverse and include, but are not limited to, neurotoxic and carcinogenic actions [36–38]. Arsenic is a metalloid that would be associated with insecticide containers discarded in the informal enterprises. In organic arsenic is considered carcinogenic and is related mainly to lung, kidney, bladder, and skin disorders [36]. The toxicity of arsenic in its organic form has been known for decades under the following forms: acute toxicity, subchronic toxicity, genetic toxicity, developmental and reproductive toxicity [39], immunotoxicity [40], and biochemical and cellular toxicity [18, 32].

The solid wastes generated in the enterprises such as lead and zinc batteries, detergent containers, and PVC contain cadmium which derives its toxicological properties from its chemical similarity to zinc. Cadmium accumulates in the human body affecting several organs that include the liver, kidneys, lungs, bones (osteomalacia; osteoporosis), the placenta, brain, and the central nervous system. Other types of damage that have been observed include reproductive and development toxicity and hepatic, haematological, and immunological effects [38]. Discarded batteries, alloys, and petroleum additives associated with the informal enterprises are linked with the heavy metal lead which has no essential function in the human body.

- Toxic waste is capable of causing injury or death through injection, inhalation, or skin absorption; some can cause cancer, genetic mutation, and foetal harm.
- Flammable/combustible wastes can be easily set on fire.
- Corrosive waste can burn and destroy living tissue or other materials when brought into contact with them.

Once in the bloodstream, lead is primarily distributed among blood, soft tissue, and mineralising tissue and children are particularly sensitive to this metal because of their more rapid growth rate and metabolism, with critical effects in the developing nervous system [37]. Mercury would be associated with containers of seed preservatives, fungicides, pharmaceuticals, and batteries discarded in the informal enterprises and it is one of the most toxic heavy metals in the environment. Thus far the disposal of e-waste with the rest of the municipal solid waste may result in negative impacts on the environment such as groundwater contamination by lead leaching and high concentrations of lead in lead leachate. When e-waste is burnt in incinerators, heavy metals become concentrated in the ash, limiting its disposal and reuse options [15]. Since most of the plastic materials in e-waste contain flame retardants that are mainly halogenated organic chemicals, toxic organic contaminants such as dioxins and furans may be formed during incineration and exit through the stack to the surrounding areas in the form of gaseous pollutants.

The actual fate of the small quantities of hazardous waste generated in municipal solid waste is generally unknown and hence the environmental persistence of these hazardous compounds is one of the critical issues in their long-term management and this is true with regard to the hazardous waste generated in the informal sector of Gweru identified in Table 2. In the informal enterprises the hazards associated with nonpersistent organic waste emanating from containers of oil, some solvents, biodegradable pesticides, waste oils, and most detergents cause toxicity problems to the environment and biota. Persistent organic wastes such as some pesticides are associated with immediate toxic effects (acute and subacute) resulting in long-term chronic toxicity and the transportation of organic waste from the source can result in widespread contamination and bioconcentration in the food chain.

### 3.2. Health and Safety Problems Affecting Enterprise Operators and Waste Workers

#### 3.2.1. Overview of Health and Safety Hazards

**Figure 2:**
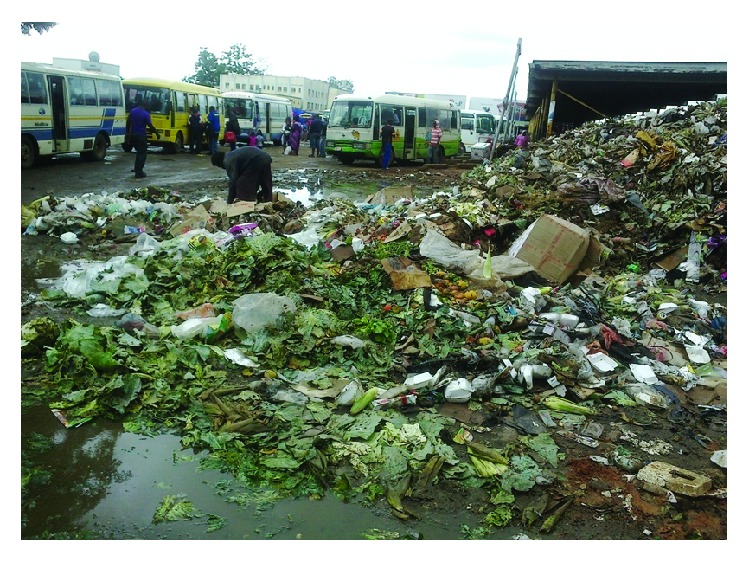
Open dumping of waste at Kudzanai market is a health hazard.

Occupational health concerns emanating from solid waste in the informal enterprises relate to the infestation of areas used for storage and disposal of solid wastes with vermin and insects that serve as potential disease vectors (Figure 2). During focus group discussions and questionnaire interviews with enterprise operators a number of waste related problems were reported. The problems identified included disease transmitting insects such as flies and cockroaches and increasing populations of rodents and odours. The Provincial Environmental Health Technician in the Ministry of Health and Child Welfare and the Senior Environmental Health Officer in the Gweru City Health Department confirmed that the problems of disease transmitting insects were attributable to the indiscriminate dumping of refuse. Enterprise operators reported that the waste related problems were attributed to noncollection or erratic collection of waste and the lack of adequate temporary storage facilities.

Open space dumping in the backyards of enterprises as well as improvised pit dumping has provided fertile grounds for breeding of disease transmitting insects such as the two-winged fly (Diptera) and cockroaches (Dictyoptera). The most important fly species from the point of view of pathogen transmission observed in the enterprise dumping areas were the housefly (*Musca domestica*) and a species of the tropical green blowfly (*Chrysomya*).* Musca domestica* breed on solid, moist, and fermenting organic matter and can develop in less than two weeks after the eggs are laid over a temperature range of 20°C–30°C [12].

This is a common phenomenon in the enterprises especially during the wet season. Cockroaches are usually attracted by the moisture in waste streams and are potential carriers of faecal pathogens. In confirming these problems the Senior Environmental Health Officer in the city of Gweru revealed that

*“flies and cockroaches breeding and feeding on the indiscriminately dumped solid waste carry particles of waste from place to place. Flies spread enteric infections such as diarrhoea, typhoid, dysentery, eye infections and skin infections such as cutaneous ephthera [sic] and yaws and incidents of such diseases as diarrhoea have occurred in the informal enterprises. These incidents are common during rainy seasons when fly populations increase and when collections are erratic due to logistic problems. The conditions at Kudzanai market as well as at Kombayi market are particularly worrying during the rainy season when uncontrolled dumping can result in unsightly heaps of waste and this is detrimental to human health.” *

Increasing rat populations were reported by 69% of the enterprise operators especially in those enterprises where waste is disposed of in open pits. The rats are such a menace and have the potential of spreading flea-borne disease and plague. Though such diseases have not yet occurred in the enterprises, they need to be guarded against as the rat populations continue to increase.

In those enterprises with an unreliable collection system, burning of combustible solid waste such as paper, plastic rubber, and textiles waste is also a common disposal method. Hot ashes which are added to combustible refuse pose a great danger to the inhabitants adjacent to the enterprises since this results in uncontrolled fires. In most cases the fires start with the objectionable practice of open burning of waste and the smoke from the burning refuse is an environmental nuisance to surrounding residents. It has also been observed that waste management procedures in developing countries are characterised by a dominance of manual handling tasks [7]. The waste generated in the informal enterprises exposes those involved in the collection and recycling to a diversity of occupational health hazards that might not be easily treated due to limited access to healthcare facilities. Exposure to occupational hazards in terms of waste management is defined by the properties of the waste, the management task (collection, transport, and recycling), and the applied procedures and technologies. Waste collection from the informal sector also involves carrying heavy loads and rotting organic waste or waste contaminated with pathogens and/or hazardous substances is handled. The occupational hazards associated with these tasks are shown in Table 3.

The waste handlers in the enterprises have shown a high risk of muscular-skeletal disorders such as low back pain and elbow/wrist pain twice as often as the control group due to handling heavy loads. Furthermore, the repetition of similar movements of hands and arms when grabbing and disposing waste containers causes joint problems as also observed by Yang et al., 2001; Cimino [41]; and Poulsen and Midtgard [42] in their studies. The risks associated with solid waste management in the informal enterprises can thus be divided into the following categories: occupational accidents, physical risks, chemical risks, ergonomic risks, psychological risks, and biological risks. The health risks either to the worker directly involved or to the enterprise operators and nearby residents are caused by many factors that include the following:

- The nature of raw waste, its composition (e.g., toxic, allergic, and infectious substances), and its components (e.g., gases, dusts, leachates, and sharps).
- The nature of waste as it decomposes (e.g., gases, dusts, leachates, and particle sizes) and their change in ability to cause a toxic, allergic, or infectious health response.
- The handling of waste (e.g., shovelling, lifting, equipment vibrations, and accidents).
- The processing of wastes (e.g., odour, noise, vibration, accidents, air and water emissions, residuals, explosions, and fires).
- The disposal of wastes (e.g., odour, noise, vibration, stability of waste piles, air and water emissions, explosions, and fires).

The health hazards associated with waste management in Gweru according to records from the Gweru City Council's Health Department are summarised in Table 4.

An interview with a health authority in the Gweru City Council confirmed the statistics shown in Table 7 which revealed that 40% of waste collectors who were referred to Gweru Provincial Hospital suffered cuts and punchers while 16% suffered from sprains. Eye injuries were mainly due to dust and smoke from the fires at the dumpsite. The official also indicated that there were no active vaccination programs for workers due to low financial allocation to the health sector by the national fiscus, although she quickly pointed out that injections were administered at the time of occurrence. She indicated that a single rabies injection/vile can cost up to US$100. Back and shoulder injuries are aggravated by lack of specialised rehabilitation equipment at the hospital. Truck injuries have the lowest incidences at 2% but when they occur they are highly life-threatening.

Table 5 shows the number and percentage of occupational injuries among workers in the cleansing section of the Gweru City Council Health Services Department, by injury type and cause from 2011 to 2012 according to statistics from the Human Resources Department.

#### 3.2.2. Mechanical Hazards

The common mechanical hazards affecting waste workers in the informal enterprises include cuts from sharp items (razor blades, glass cutlets, and metal pieces) and needle pricks from dressmaking enterprises. There is also the risk of tetanus resulting from rusty wires and scrap metals. Observations revealed that workers are also at risk of being electrocuted from naked wires, wrong wiring connections, traumatic injuries from sharp objects, burns from electric sparks during electrical fixing, dust from carpenters and grind mills, noise from welders and milers, and exposure to heat and ultraviolet radiation from welding. Health hazards also emanate from infections caused by biological agents, especially virus infections such as hepatitis B/C. Tetanus infection is also a serious concern since some of the workers are not vaccinated and the wounds are not treated adequately due to a lack of hygiene and the necessity to resume work immediately in order not to lose income. Other mechanical risks include bruises from hitting equipment, fractures, and contusions evoked by falling from unsecured platforms of trucks. However, closely connected with waste collection are cuts from sharp items from waste generated in the informal enterprises as well as falling accidents from small platforms of waste collection trucks. The mechanical safety and health problems associated with solid waste management in the informal enterprises were succinctly explained by a municipal waste worker who was busy collecting waste at Monomotapa:

*“mechanical hazards associated with solid waste generated and disposed in the informal sector include piercing, scraping and bruising by scrap metals, old wires and vehicle shells resulting in wounds from contact with sharp waste. Hazards like broken bottles, liquid fires at fuelling depots, residual fires at landfills, bins with jagged edges and compactors pose safety hazards to us employees. Broken bottles, glasses and other sharp objects impale our already worn out gloves thus exposing us to cuts and bruises which may lead to diseases like tetanus, dermatitis and may eventually fester into septic wounds. We also do not have adequate protective clothing to protect ourselves especially face masks, gloves and overalls.”*

**Figure 3:**
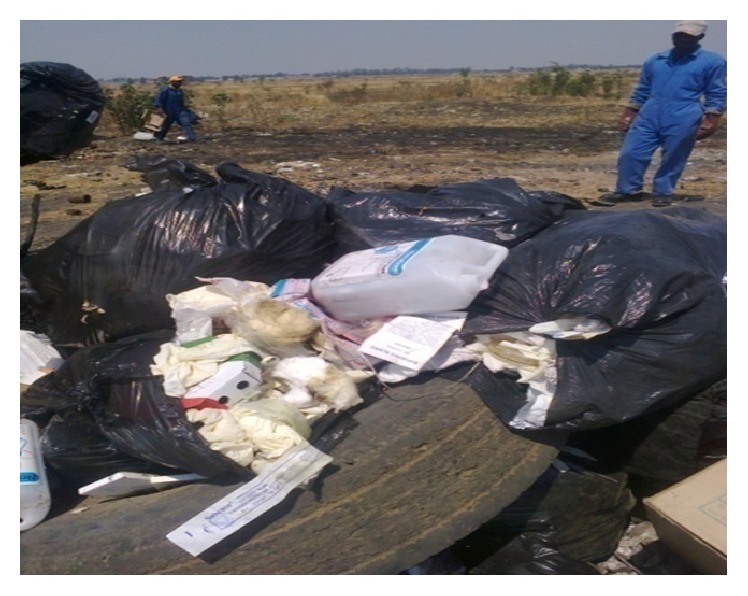
A waste worker about to burn hazardous waste without a face mask.

There are various methods used by the Gweru City Council to prevent injuries and these include the use of personal protective equipment (PPE), personal protective clothing (PPC), and safety warnings. Safety related injuries are the major problem in most sections. PPC such as dust masks and respirators are used to deal with problems of high levels of dust and smoke. However, landfill workers and bin loaders complained that the material used to make the masks is not very effective since they are facing respiratory difficulties during the time of waste burning. Some of the masks do not fit to faces since they do not have room for adjustment; hence some workers would rather operate without masks, a move that may be detrimental to their health and most of the time most workers do not have the masks since they are usually in short supply (Figure 3). Work-suits and safety shoes are also used as a way of protecting the body from harmful objects. Furthermore, ear plugs are used in areas with high levels of noise. Working in areas with high levels of noise can cause long-term effects to the human audio system.

#### 3.2.3. Ergonomic Hazards

**Figure 4:**
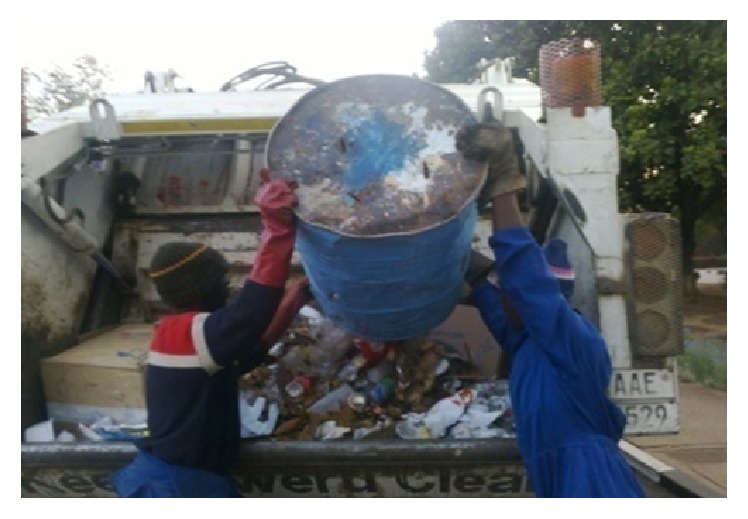
Waste collection involves manual handling tasks.

Ergonomic hazards in the informal enterprises result from carrying or lifting heavy loads, repetitive movement and work, that is, shovelling, muscular-skeletal disorders resulting from handling heavy containers, heat stress resulting from exposure to excessive temperatures, and hearing loss due to too much exposure to excessive noise. Collection and sorting operations require repeated lifting and twisting motions which are common sources of musculoskeletal injuries including repetitive strain injuries. Collection workers must lift, twist, and dump heavy bins and bags and during curbside sorting the lifting can exceed guidelines recommended and hence is likely to cause harm (Figure 4).

Manual sorting tasks often require reaching, lifting, and twisting and this can cause workers pain, soreness, general fatigue, tendonitis, and musculoskeletal injuries of the feet, arms, shoulders, hands, wrists, and lower and upper back. Observations showed that garbage workers experienced a high incidence of repetitive strain injuries because of repeated flexing and twisting motions, further noting that waste collection workers are usually inadequately trained and prepared for the fine motor activities required for curb side sorting hence exposure to ergonomic hazards (Table 6). It is the awkward postures, forceful exertions, static loading, extended reaches, deviated wrist hand and arm postures, and contact stress which present major ergonomic hazards.

A total of 32 waste collectors were interviewed on healthy ergonomics behaviour; 29 of them are male and aged between 18 and 50 years. It became clear that most men had some insight into the occupational hazards of their workplaces but generally lacked thorough factual occupational health and safety knowledge. The respondents were able to mention certain safety related occupational health risks but did not consider these hazards to be dangerous to their health or capable of causing disease. For example, the waste collection crews in Mkoba and Ascot considered their trade to be dangerous but could not explain the health effects that were related to the job. The level of awareness regarding the major areas of ergonomics was found to be low among the collection crew members who operated in the informal enterprises when compared to the office workers as shown in Table 7.

#### 3.2.4. Chemical Hazards

In identifying the health impacts of chemical and biological agents in the informal sector, the possible obstructing factors include the following: the long period before the effect becomes manifested, the multiplicity of causes of diseases (which makes it difficult to distinguish occupational diseases from diseases caused by, e.g., unhygienic living conditions); the lack of knowledge mechanisms involved in the pathogenesis of human chronic diseases; and a wrong classification of diseases. There is high danger of skin and blood infections resulting from direct contact with these liquids and from infected wounds intoxication and skin irritation resulting from contact with small amounts of hazardous chemical waste. Residues of hazardous chemicals in recyclable containers and their gaseous emissions pose hazards to workers involved in the collection, sorting, and washing processes. Chemicals that pose risks include chlorine, fluorine, paper beaching, deinking, pulping agents, plastic additives and equipment cleaning solvents, and insecticides and herbicides. Contact with skin or inhalation or even ingestion of these chemicals can cause dermatitis, disorder to the central nervous system, and possible liver and kidney damage. Exposure to fumes from heated metals can produce metal fume fever which is a flu-like condition. Exposure to chemicals can also cause irritation to the skin and respiratory tract and potential damage to the liver and central nervous system. Inhalation of metal, glass, paper, or plastic dust from shredding, demagging, and detinning can cause or aggravate chest discomfort, bronchitis, or asthma. Acute exposure to metal dust may cause irritation of the upper respiratory system and eventually severe pulmonary irritation. Chronic exposure to some heavy metals may cause cancer and adverse effects to the central nervous gastrointestinal system. Disposal of old batteries and electronic and electrical appliances such as cell phones, radios, computers, televisions, digital satellite decoders, and fluorescent tubes may pose danger as these contain toxic substances such as mercury, lead, and cadmium.

Motor mechanics and welders at Shamrock Park, Monomotapa, Ascot, and Mkoba use paraffin, paint, and solvents such as benzene and methylated spirit and there is high danger of skin and blood infections resulting from direct contact with these liquids. Scrap batteries removed from vehicles have the potential of corroding clothes, causing blisters, and fire outbreaks due to the acid containers. Scrap metal from welding shops and garages is hazardous since people experience cuts when collecting and disposing waste materials. Rusting metals have the potential of causing tetanus in people. Empty bottles of toxic chemicals are dangerous to children who play with these and poisoning may occur through ingestion, absorption, and inhalation of gases in empty containers.

#### 3.2.5. Biological Hazards

Biological hazards associated with waste generated and disposed of in the informal sector enterprises include water borne diseases resulting from flies and mosquitoes breeding in dumping sites around the enterprises. Rabid dogs scrambling in bins may result in bites that cause rabies and rodents may also spread disease. Dermal and blood infections may result from direct contact with waste and from infected wounds, zoonosis due to bites by wild or stray animals feeding on waste, and enteric infections transmitted by insects. Leaching of toxic matter in areas close to the dumps leads to contamination of water sources resulting in diarrheal diseases. Workers may be infected by biological agents such as bacteria and viruses that contaminate waste, which are usually formed from the decomposition of matter and result in infections. Cuts or puncture wounds from broken glass, metal edges, or needles become the site of infection following exposure to bacteria and viruses and the infections include hepatitis B, fungi, or parasites. Common health problems associated with exposure to certain bacteria, fungi, and viruses include contact dermatitis infections, diarrhoea, and skin diseases. Long-term occupational exposure to contaminated air in composting operations can include allergic responses such as asthma, chronic bronchitis, and hay fever. Other symptoms in waste workers include chills, irritation of eyes, nose, and upper respiratory tract, nausea, headache, chest tightness, and feeling of influenza. Workers in paper sorting operations have the highest incidence or chances of lung infections compared to all other waste workers and this is a result of high levels of organic dust and endotoxins (poisonous substances produced by bacteria in the air). Water-borne diseases are also biological hazards emanating from flies and mosquitoes breeding in dumpsites and causing malaria. Dermal and blood infections from direct contact with waste and from infected wounds, zoonosis resulting from bites by stray animals feeding on waste, and enteric infections transmitted by insects are the other biological hazards. It has been documented that waste workers experience higher incidents of diarrhoea, viral hepatitis, and higher incidents of obstructive and restrictive respiratory disorders than control groups and suffer from dog and rat bites, skin diseases, and jaundice [34, 43]. Some of the problems that were reported by the authorities in the city of Gweru as emanating from waste generated in the enterprises are like common cold, cough, bronchitis, bronchial asthma, tuberculosis, and other respiratory problems. However, other authors such as Van Eerd [34] and Porta et al. [44] have noted that it is difficult to prove a direct link between these diseases and the waste occupation.

## 4. Discussion and Conclusion

Occupational exposure in the case of solid waste management activities in the informal enterprises of Gweru is influenced especially by the properties of the waste and secondly by the management task which involves collection and disposal as well as the applied procedures and technologies. Solid waste management procedures in the informal sector of Gweru are characterised by a dominance of manual handling tasks. Collection involves carrying heavy loads and rotting organic waste or waste contaminated with pathogens and/or hazardous substances. The working conditions and properties of the waste expose workers involved in collection and disposal of waste to a diversity of occupational safety and health hazards that might not be treated adequately due to limited resources.

**Figure 5:**
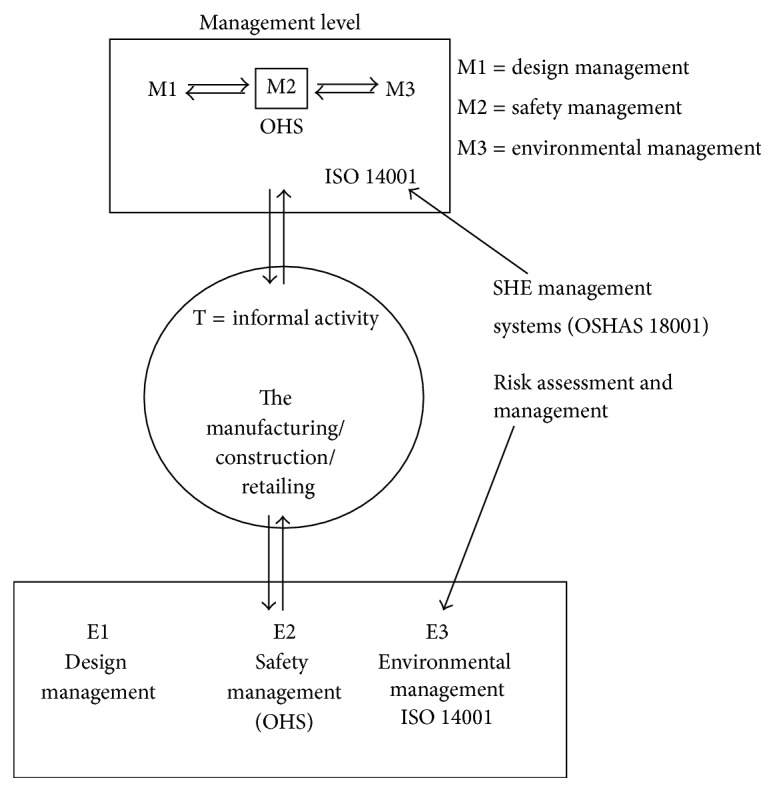
An idealised solid waste management model for the informal sector of Gweru.

A holistic view of waste management implies integrating the waste management system into the informal enterprises activities and the Gweru Municipality as an organisation since this incorporates occupational safety and health aspects (see Figure 5). For the manufacturing and construction enterprises in Monomotapa, Shamrock Park, Mkoba, and Ascot there would be need to take into account the waste management issues as an integral part of the design activity. T would represent the process such as construction and manufacturing, while E would represent an aggregate of the base level process design activity B1 and another base level activity E2 which both refine T by specifying cycle by cycle its attributes with an aim to end up with an acceptable* performance* of T assessed against a predefined set of* performance* criteria. E2 refers to the SHE system taking into account safety, health, and environmental issues of the activities. Waste workers in the informal enterprises of Gweru experience a number of adverse health and safety effects and these include higher incidents of diarrhoea, viral hepatitis, higher incidents of obstructive and restrictive respiratory disorders, and dog and rat bites, skin diseases, and jaundice. There are also higher incidents of muscular-skeletal disorders affecting the waste collectors such as low back pain and elbow/wrist pain and joint problems which arise from the repetitive movements of hands and arms when grabbing and disposing waste containers. The common mechanical hazards in the informal enterprises of Gweru include cuts from sharp items such as razor blades, glass cutlets, and metal pieces. Workers are thus exposed to the risk of infections caused by biological agents, especially virus infections. Infections such as hepatitis B/C and tetanus are a major concern since workers are rarely vaccinated and wounds are not treated adequately due to a lack of hygiene and the desire to resume work immediately so as not to lose income. Mechanical risks experienced by waste workers in Gweru include bruises from hitting equipment, fractures, and contusions evoked by falling from unsecured platforms of trucks.

**Figure 6:**
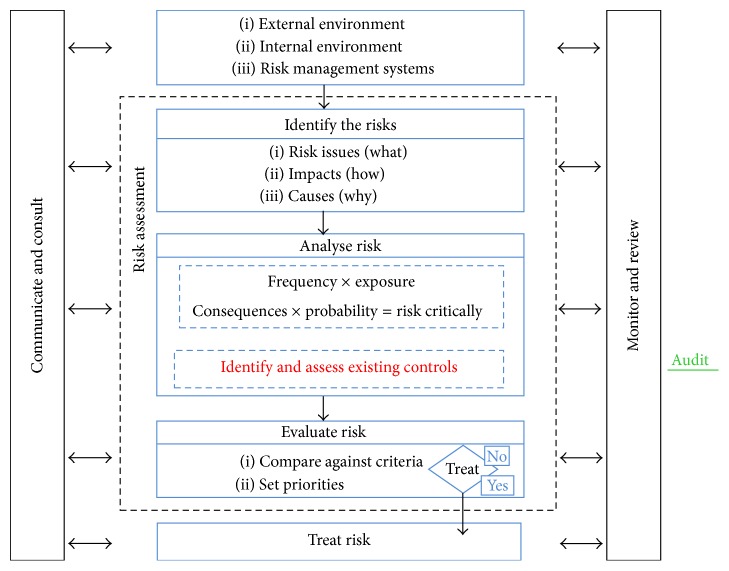
The risk assessment and management process.

Since safety, health, and environmental management systems are a vital component of the waste management model shown in Figure 5, risk assessment therefore becomes imperative in determining and evaluating the risks posed by the working conditions of the waste workers. Risk assessment is a systematic examination of all aspects of work and it considers what could cause injury or harm, whether the hazards could be eliminated, and what preventive or protective measures should be put in place to control the risks [16]. Risk assessment is the starting point of the risk management process. Undertaking risk assessment would enable the municipality of Gweru and the enterprise operators to understand the action necessary to improve workplace occupational health and safety. The ultimate objective is to decide on an action plan designed to establish the control of risk and to ensure that risk control remains effective. Risk assessment directly relates to the actual techniques and procedures in detecting what hazards could cause injury or long-term health impacts [7].  Figure 6 shows the main elements of the risk assessment and management process. The focus group discussions with waste workers and interviews with waste authorities in Gweru showed indeed that transfer mechanisms of waste from temporary waste disposal receptacles into municipal receptacles needed urgent attention.

The risk assessment survey also showed that the waste management conditions in the informal sector enterprises were hazardous. Waste collection involved manual handling of plastic and metal bins and this was associated with a number of ergonomic hazards as discussed in the previous sections. Some of the roads, especially in Mkoba, Ascot, and Monomotapa high density suburbs, were rough and unpaved and hence posed risks in the form of road accidents. Waste was also sometimes strewn down the streets from the collection vehicles. In all the enterprises including those in Monomotapa and Shamrock Park, sharp items such as razor blades, glass cutlets, and syringes as well as hazardous substances such as broken batteries and leaking solvent containers can be found. In cases where plastic bags were used for collecting solid waste, the thin permeable material posed dermal exposure because hazardous substances, microorganisms, and sharp items also injure workers when handling the waste bags with bare hands. There is inadequate and improper personal protective clothing (PPE) as evidenced by the torn or makeshift protective clothing such as the gloves worn as protection by the workers.

It was also revealed through risk assessment that most of the waste workers as well as enterprise operators had been affected by cuts and skin rashes that were caused by substances and insects associated with the disposed-of solid waste. The open wounds were also at risk of being infected by tuberculosis in such unhygienic working conditions. It has been observed by Bleck and Wettberg [7] that hepatitis B infections can occur when the cuts are caused by razor blades or syringes which are disposed of in the ordinary waste stream. Dust is generated in quite visible amounts in informal sector enterprises especially at Monomotapa, Kombayi market, Ascot, and Mkoba. This was during the pouring of waste into collection bags and also during the transfer of waste into containers. Dust constitutes a major hazard because of its contribution to inhalation; exposure to biological agents and bronchial asthma, cough, and other respiratory problems may result. The ergonomic hazards are exacerbated by the carrying and emptying of heavy unstable waste bags and this is a major health hazard among the female workers.

The safety interventions in Gweru are complicated by the fact that solid waste collection is undertaken through labour intensive systems and hence workers experience high physical loads and inadequately stored waste. In the low-tech waste management sector of the city of Gweru, occupational safety and health intervention is often equalled with the supply of personal protective clothing. This has been proven to be one of the least effective measures due to the demand for correct application, infrequency of supply, and inadequate materials as also in studies undertaken elsewhere by KENAO. Officials from the Health Department of the city of Gweru indicated that their safety interventions included mainly the provision of PPE. The Environmental Health Officer in the city of Gweru indicated that “we provide our waste collection crew with PPE to protect themselves against occupational hazards associated with the collection and disposal of solid waste and this has proven to be effective through the years.” Van Eerd [34], however, notes that health officials may not be aware that protective devises are among the least effective safety interventions and that the long distribution intervals, especially for masks, rendered the supply itself absurdum. Usually even when workers are supplied with the protective equipment, they normally do not use it as a result of lack of awareness as well as their low social status. A sustainable solution to increase occupation safety and health among the workers would be the adaptation of workplace and process design.

Improving the occupational safety of waste workers is thus a crucial step to increase their social welfare. This can only be done in an efficient manner by firstly identifying the actual occupational risks associated with solid waste management activities. This is vital in the quest to apply a hierarchy for exposure control measures as initiated by the Council Directive 89/391/EEC of June 1989. This entails eliminating the hazard at its source, for example, substituting hazardous chemicals or omitting burdensome work steps and hence rendering additional work steps unnecessary and it is the most efficient precaution. Technical measures are also vital and these involve safer equipment and are more preferable to individual measures such as personal protective equipment and training in proper behaviour. These do not eliminate the hazard per se but only provide a barrier between the hazard and the worker at the ultimate point. This is the STOPP principle:

1. S: substitution of hazardous process or material.
2. T: technical measures.
3. O: organisational measures.
4. P: personal protective equipment.
5. P: personal behaviour.

The lack of a comprehensive waste policy that is packaged to deal with safety, health, and environmental management issues in Zimbabwe has compromised effective solid waste management in the informal sector. There is lack of consensus on what constitutes solid waste, its characteristics, and how the waste should be managed and this has resulted in the municipalities having no proper guidelines over the organisation of sustainable solid waste management in the informal enterprises.

## Figures and Tables

**Table 1:** Average composition of hazardous solid waste generated in the informal enterprises per week (% by weight).

Kudzanai/Kombayi	Shamrock Park Monomotapa	Mkoba	Ascot
*Containers of* (i) Paint (ii) Outdated medicines (iii) Toilet bowl cleaners (iv) Hair waving lotions (v) Shampoos (vi) Nail polish (vii) Disinfectants (viii) Wood preservatives (ix) Scouring powders (x) Spot removers *Sharps* (i) Broken glass (ii) Opened tin cans *WEEE* (i) Cell phones (ii) Irons (iii) Calculators (iv) Air conditioners (v) VCRs and DVDs	*Containers of* (i) Motor oil (ii) Paints (iii) Spot removers (iv) Diesel and petrol (v) Brake fluid (vi) Glass cleaners (vii) Drain cleaners (viii) Silver polish (ix) Furniture polish (x) Spot removers (xi) Carpet cleaners (xii) Upholstery cleaners (xiii) Photographic material (xiv) Pool cleaners (xv) Laboratory chemicals (xvi) Paint solvents *Sharps* (i) Scrap metal (ii) Zinc pieces (iii) Cable strip (iv) Broken glass (v) Pieces of wire (vi) Opened tin cans *WEEE* (i) Large home appliances (ii) Small home appliances (iii) IT and telecom equipment (iv) Consumer equipment *Others* Plastic Acid lead Batteries	*Containers of* (i) Scouring powders (ii) Shoe polish (iii) Furniture polish (iv) Glass cleaners (v) Shampoos (vi) Outdated medicines (vii) Pesticides (viii) Insecticides (ix) Disinfectants (x) Wood preservatives (xi) Cosmetics (xii) Hair lotions (xiii) Toilet bowl cleaners (xiv) Laboratory chemicals (xv) Ant and roach killers *Sharps* (i) Broken glassware (ii) Nails (iii) Scrap metal (iv) Needles (v) Pieces of wire (vi) Opened tin cans *WEEE* (i) Home appliances	*Containers of* (i) Wood preservatives (ii) Toilet bowl cleaners (iii) Disinfectants (iv) Laboratory chemicals (v) Scouring powders (vi) Shampoos (vii) Hair waving lotions (viii) Paints (ix) Glass cleaners (x) Toilet bowl cleaners (xi) Outdated medicines (xii) Ant and roach killers *Sharps* (i) Broken glassware (ii) Nails (iii) Needles (iv) Opened tin cans *WEEE* (i) Large home appliances (ii) Small home appliances (iii) IT and telecom equipment (iv) Consumer equipment

* 1.8% by weight*	*3.87% by weight*	*2,46% by weight*	*2,22% by weight*

Source: field survey (2015).

**Table 2:** Concerns associated with hazardous municipal solid waste.

Product	Concern
*Cleaners*	
Abrasive cleaning powders	Corrosive/toxic
Aerosols	Flammable/toxic
Furniture polish	Flammable/toxic
Glass cleaners	Irritant/toxic
Outdated medicines	Toxic
Shoe polish	Flammable
Spot remover	Flammable/toxic
Toilet bowl cleaner	Corrosive
Carpet cleaner	Flammable/toxic
*Personal care products*	
Hair waving lotion	Toxic
Medicated shampoos	Toxic
Nail polish remover	Toxic/flammable
*Automotive products*	
Brake transmission fluid	Flammable/toxic
Car batteries	Corrosive/toxic
Diesel and petrol	Flammable/toxic
Waste oil	Flammable/toxic
*Miscellaneous*	
Batteries	Corrosive/toxic
Pesticides, herbicides, and fertilisers	Toxic/flammable
Insecticides	Toxic

Source: Tchobanoglous et al. [12].

**Table 3:** Occupational hazards affecting waste workers in the informal enterprises.

Hazard	Task
Muscular-skeletal disorders	Lifting and carrying heavy loads and pushing pushcart
Biological agents	Handling of organic waste, handling contaminated materials, and working in contaminated environment (mould, dirt)
Hazardous substances	Working with mixed waste
Mechanical hazards	Unintentional contact with sharp items and working near moving parts of machinery/vehicles
Noise/machinery	Working near heavily frequented roads and in the vicinity of loud vehicles (enterprise workshops such as carpentry, metal work, and engineering)
Vibration	Pushing vehicles on uneven ground
UV/IR radiation	Working in the sun
Electrical risks	Taking waste from workshops
Psychological burden	Working with waste and disrespect of society

**Table 4:** Waste management related hazards identified by the Health and Sanitation Department of the city of Gweru.

Department	Hazards	Impact(s)
Health and Sanitation	Biological	Hepatitis B Cholera Diarrhoea Respiratory diseases causing flu to employees Nasal irritation and nausea Eye irritation
	Physical	Hearing loss High temperatures in working environments causing dizziness Hypothermia from low temperatures Frost bites and flu
	Ergonomic	Musculoskeletal injuries (MSIs) Repetitive strain injuries (RSIs) Long-term back pains and eventually strokes

	Safety	Limb loss from compactor hydraulics Acidic corrosion Burning at dumpsites after explosion of pressurized containers like aerosols and so forth

	Chemical	Cancers from carcinogens Disorders to the central nervous system (CNS) Possible lung, kidney, or liver damage

Source: Gweru City Council (2014).

**Table 5:** Occupational injuries among workers in the cleansing section.

Type	Risk factor	Number	(%)
Cut on hand, finger, thumb, or foot	Broken glass or sharp objects	27	(40)
Sprained ankle or wrist	Improper lifting or throwing technique or running and disembarking from vehicle	11	(16)
Eye injury	Dust, liquid, chemicals, or smoke	8	(12)
Shoulder injury	Contact collision	8	(12)
Knee injury	Contact collision, slip, or fall	5	(8)
Laceration of leg or finger	Dog, rat, scorpion, or snake bites	4	(5)
Sharp back pain	Excessive effort in lifting	3	(5)
Trunk injury	Run over by truck	1	(2)
*Total*		*67*	

Source: Gweru City Council Human Resources Department (2014).

**Table 6:** Distribution of health complaints (%) related to poor ergonomic practices.

Health complaints	Ascot	Monomotapa	Shamrock Park	Mkoba	Kudzanai	Kombayi
Chronic back pain	51	53	48	46	36	21
Chronic neck pain	11	67	57	66	61	41
Chronic shoulder pain	69	56	54	52	56	53
Repetitive strain injuries	76	71	73	75	86	69
Repetitive motion injuries	76	72	72	73	81	70
Sprained arms and knees	66	43	49	58	66	68
Exposure to dust	89	86	87	81	77	81
Electric shock	2	21	38	14	5	2
Eye injury	25	48	68	66	51	56
Excessive noise	13	26	32	31	8	7
Excessive heat	19	13	23	24	22	25

Source: field survey (2015).

**Table 7:** Level of awareness (%) concerning major areas of ergonomics.

Spatial location	Human factors of engineering (Prevention of accidents)	Work physiology (Prevention of fatigue)	Occupational biometrics (Prevention of musculoskeletal disorders)	Use of anthropometric data (Postures and work)
Shamrock Park	43	53	41	66
Monomotapa	27	20	36	11
Kudzanai	16	18	23	26
Kombayi	11	13	22	22
Ascot	18	29	16	34
Mkoba	7	38	31	19
